# An Unappreciated Cause for the Falling off of Prescriptions

**Published:** 1916-04

**Authors:** 


### An Unappreciated Cause for the Falling Off of Prescriptions

With all allowance for economic and frictional factors, we think that the Pharmaceutic Profession fails to realize a very important reason for the diminished volume of prescriptions. The old fashioned four to six ingredient prescription, with its active drug, corrigent, diluent and flavor, is practically obsolete. This is nobody's fault, least of all the druggists. The homoepaths had a good deal with teaching us to use one drug at a time. Various specialists have shown the superior efficacy of local applications, even pretty deep within the body cavities, instead of relying on the selective action or chance penetration of smaller quantities of much larger, perhaps dangerous aggregate doses. Chemists have given us tasteless substitutes for nauseous drugs, manufacturers have given us various devices for concealing disagreeable tastes, either means being superior to mixing supposedly agreeable with certainly disagreeable flavors.

The "N.F." exists largely for the sake of resisting the current of modern therapy. If a drug deserves formal listing at all, let it be in the U. S. P. A stock list of formulae tends to discourage rational variation of dosage and to weaken the ability to prescribe extemporaneously. It is also an obstacle to the achievement of simple, decimal formulae.

But there is much to be said in favor of the "N.F." and we shall be glad to publish this side in a later issue.

